# Synthesis and Gene Silencing Properties of siRNAs Containing Terminal Amide Linkages

**DOI:** 10.1155/2014/901617

**Published:** 2014-03-26

**Authors:** Maria Gaglione, M. Emilia Mercurio, Nicoletta Potenza, Nicola Mosca, Aniello Russo, Ettore Novellino, Sandro Cosconati, Anna Messere

**Affiliations:** ^1^Dipartimento Scienze e Tecnologie Ambientali, Biologiche e Farmaceutiche, Seconda Università degli Studi di Napoli, Via Vivaldi 43, 81100 Caserta, Italy; ^2^Dipartimento di Chimica Farmaceutica e Tossicologica, Università “Federico II,” Via D. Montesano 49, 80131 Napoli, Italy

## Abstract

The active components of the RNAi are 21 nucleotides long dsRNAs containing a 2 nucleotide overhang at the 3′ end, carrying 5′-phosphate and 3′-hydroxyl groups (siRNAs). Structural analysis revealed that the siRNA is functionally bound at both ends to RISC. Terminal modifications are considered with interest as the introduction of chemical moieties interferes with the 3′ overhang recognition by the PAZ domain and the 5′-phosphate recognition by the MID and PIWI domains of RISC. Herein, we report the synthesis of modified siRNAs containing terminal amide linkages by introducing hydroxyethylglycine PNA (*heg*PNA) moieties at 5′, and at 3′ positions and on both terminals. Results of gene silencing studies highlight that some of these modifications are compatible with the RNAi machinery and markedly increase the resistance to serum-derived nucleases even after 24 h of incubation. Molecular docking simulations were attained to give at atomistic level a clearer picture of the effect of the most performing modifications on the interactions with the human Argonaute 2 PAZ, MID, and PIWI domains. This study adds another piece to the puzzle of the heterogeneous chemical modifications that can be attained to enhance the silencing efficiency of siRNAs.

## 1. Introduction

RNA interference (RNAi) has come into the limelight in the antisense world following the discoveries of Mello and colleagues [1] that double-stranded RNAs (dsRNAs) can elicit potent degradation of targeted mRNA sequences in* C. elegans* and in mammalian cells [2, 3]. The active components of the RNAi are small interfering RNAs (siRNAs), 21-22 nucleotides long dsRNA. These short species are naturally produced by Dicer-mediated cleavage of larger dsRNAs and they contain a 2 nucleotide (nt) overhang at the 3′ end, a 5′ phosphate and a 3′-hydroxyl group [2, 4]. Synthetic siRNAs can also be introduced into cells in order to experimentally activate RNAi [2]. siRNA duplexes with 5′-hydroxyl ends are rapidly phosphorylated in cells by the cellular kinase Clp1 [5], then the siRNA strand with the thermodynamically less stable 5′ end is preferentially incorporated as the guiding or antisense strand (AS) in the RNA-induced silencing complex (RISC) [6], while the passenger or sense strand (SS) of the siRNA duplex is cleaved by the human Argonaute 2 protein (hAgo 2) and liberated from the complex [7]. The selection of the guide strand is then based on the thermodynamic stability of the siRNA duplex ends, the strand that is always the one whose 5′ end is less tightly paired to its complement. When the siRNA is fully base paired, the local thermodynamic difference (thermodynamic asymmetry) between the two 5′ ends favors assembly into RISC of the strand with the lower internal stability at 5′ end.

The Argonaute proteins are, indeed, core components of RISC and are made up by PAZ, Mid, and PIWI domains. X-ray structural analysis [8, 9] revealed that the siRNA is bound at both ends: the 5′ end to the MID domain with some contributions from the PIWI domain and the 3′ end to the PAZ domain. The seed sequence is located in a narrow portion of the RNA binding groove. Endogenous pre-microRNAs, transcribed by cellular RNA polymerase II as single-stranded hairpin-loop RNAs, are also cleaved by Dicer to yield microRNA (miRNA) duplexes. These molecules are further processed as described for the siRNAs and incorporated in RISC by association of their guide strand to hAgo 2. Mature miRNAs play crucial roles in the regulation of gene expression during development and cell differentiation [10]. In addition, recent studies indicate that they are important regulators of virus-host interactions [11, 12].

Given its reliability and ease of use, RNAi has become the most widely used technology in functional genomics studies* in vitro* and in several model organisms. Nevertheless, to translate this potential into a broad new family of therapeutics, it is necessary to optimize the efficacy of the RNA-based drugs [13]. It might be possible to achieve this optimization using chemical modifications that improve, just like for antisense oligonucleotides (ASOs) [14], their* in vivo* stability, cellular delivery, biodistribution, pharmacokinetic, potency, and specificity [15]. In this respect, a rational design of effective chemically modified siRNA must consider as a general principle that the two strands of siRNA function differently and as practical hint that the nucleotides are different according to positions and nature. The 3′ and 5′ ends of siRNAs are critical determinants of their capacity to interfere with the unwinding of the duplex, of the incorporation of the siRNA into RISC, and of the rate of target cleavage and product release. The modifications in the terminal positions are considered with interest because the introduction of chemical moieties in these regions interferes with 3′overhang recognition by the PAZ domain [8, 16] and 5′-phosphate recognition by the MID domain of RISC [17, 18].

Peptide Nucleic Acids (PNAs) are oligonucleotide mimics in which the sugar-phosphate backbone has been replaced by a pseudo-peptide backbone [19]. When used in antisense constructs, PNA confers chemical and enzymatic stability and high affinity towards complementary DNA and RNA [19, 20]. Nonetheless, PNA have limited solubility and tendency to aggregate and are not easily internalized into cells, whereas oligonucleotide PNA (ON-PNA) chimeras are molecules with high solubility and increased capacity to cross biological membranes as compared to canonical PNA. Chimeric molecules in which tracts of DNA are bound to N and/or C terminus of PNA have been widely reported [21–31]. Differently from DNA-PNA chimeras, not many studies have been conducted on RNA-PNA chimeras. So far, few results have been reported for the use of RNA-PNA chimeras in RNAi, even if the advantage of mixing peptide and nucleic acids bonds has been demonstrated [27, 32–34].

**Figure 1:**
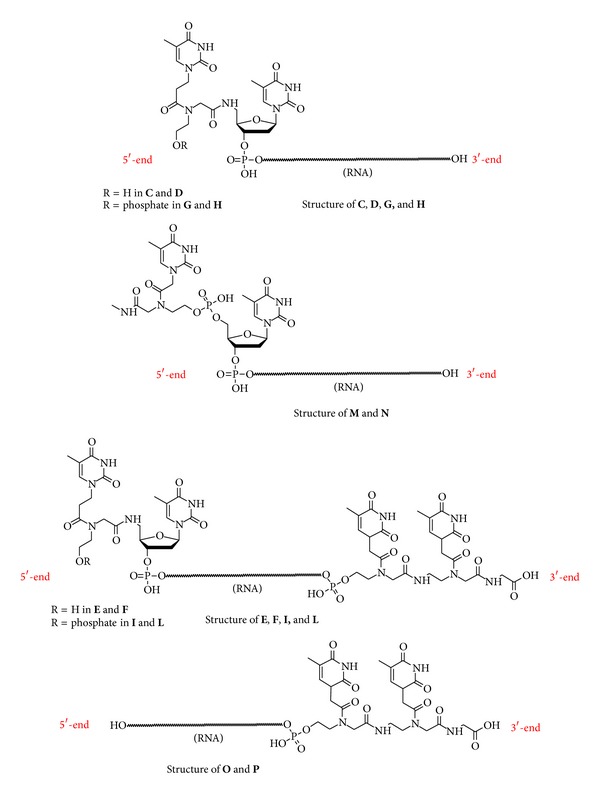
Structure of the RNA oligomers forming modified siRNAs** 2–19**.

In this paper we describe the synthesis of modified siRNAs containing terminal amide linkages (Figure 1). RNA-5′-PNA-OH (siRNA** 2–4**, Table 1) and RNA-5′-PNA-O-phosphate (siRNA** 8–10**, Table 1) were synthesized with the aim to study the impact of hydroxyethylglycine backbone (*heg*PNA) on silencing activity. The effect of* heg*PNA on both 5′ and 3′ ends of the siRNAs was also investigated by synthesizing PNA-3′-RNA-5′-PNA-OH (siRNA** 5–7**, Table 1) and PNA-3′-RNA-5′-PNA-O-phosphate (siRNA** 11–13**, Table 1). Modified siRNAs containing* heg*PNA at 5′ and at 3′,5′ ends were then compared with chimeric 3′-PNA-OH siRNAs (siRNA** 17–19**, Table 1). At last, we introduced a new modification into siRNA consisting of a* heg*PNA monomer inserted at 5′ end of RNA domain by phosphodiester linkage and having at the C-terminus a methylamide function (herein referred as “5′-capped” siRNAs,** 14–16** in Table 1). The biostability of** 2**–**19** was investigated through incubation in 100% fetal bovine serum (FBS), revealing that PNA moieties on both strands of the siRNA markedly increase the resistance to serum-derived nucleases. Gene silencing studies performed in HeLa cells highlighted that the amide linkage is compatible with the RNAi machinery when placed at 3′ end of siRNA, in some cases improving its performances. On the other hand, amide linkage dramatically decreased the interference activity when located at 5′ or at both 3′ and 5′ regions of siRNAs. Interestingly, “5′-capped” siRNAs partially restored the interfering effect. Finally, molecular docking simulations were engaged to give a picture of the interactions of the most performing modifications with the hAgo 2 protein.

## 2. Materials and Methods

### 2.1. Materials and Apparatus

SynBase CPG solid supports loading 0.040 and 0.10 meq g^−1^, 2′-OTBDMS-RNA phosphoramidite, PNA monomers, and standard RNA-synthesis reagents (Acetonitrile external wash (wash A), Amidite diluent, Activator solution, Cap A, Cap B, Acetonitrile wash B, Oxidizer were purchased from Link Technologies (Lanarkshire, Scotland). Standard PNA-synthesis reagents (PyBop activator, HOBT, Base solution, Cap solution, Piperidine, Deblock solution, DMF, wash B) were from Novabiochem. DMF external wash (wash A, 0.01% H_2_O) was from LabScan. All other reagents and solvents were from Sigma-Aldrich. Solid phase synthesis was performed on an ABI Expedite 8909 oligo synthesizer using standard and modified protocols. HPLC chromatographic analyses and purifications were performed on Nucleogel SAX (Macherey-Nagel, 1000-8/46) and RP-18 (Waters, C-18, 3.9 × 300 mm) columns using a Waters 600 Controller, equipped with the diode array detector Waters 996 and with Millennium software. For column chromatography, silica gel 60 (0.040–0.063 mm) from Merck was used. Centrifugations were performed on a Z 200 A Hermle centrifuge. Samples were lyophilized by FD4 Freeze Dryer (Heto Lab Equipment). All conjugates were analyzed by MALDI TOF mass spectrometry using a MALDI-TOF micro MX (Waters Co., Manchester, UK), equipped with a pulsed nitrogen laser (*λ* = 337 nm). All measurements were performed using the negative detection mode. All spectra were processed and analyzed using the MassLynx 4.1 software (Waters, Milford, MA USA). LC ESI-MS analyses were performed on a MSQ mass spectrometer (ThermoElectron, Milan, Italy) equipped with an ESI source operating at 3 kV needle voltage (*T* = 320°C) and with a complete Surveyor HPLC system, comprising a MS pump, an autosampler, and a PDA detector. All buffers were prepared from highly purified Milli Q water and made RNase-free by treatment with DEPC (diethylpirocarbonate) (Sigma Aldrich). UV measurements and melting curves of duplexes (1.0 *μ*M) were acquired on a Jasco V-550 spectrophotometer equipped with a Jasco ETC-505T Peltier temperature programmer using a 1 cm path-length quartz cell (Hellma). The concentrations were estimated spectrophotometrically at 90°C using the following additive molar extinction coefficient *ε* ^260^(L cm^−1^ mol^−1^),* T* = 8800,* A* = 15400,* C* = 7200,* G* = 11500, and* U* = 9900 for the natural nucleobases and* t* = 8600 for the PNA monomers. Oligomers were suspended in RNase-free annealing buffer (100 mM potassium acetate, 30 mM Hepes-KOH, pH = 7.4, and 2 mM magnesium acetate) and equimolar ratios of the sense and antisense strands were annealed to form the duplexes to a final concentration of 25 *μ*M by incubation at 90°C for 1 min and gradually cooling to room temperature. Melting curves were recorded at 260 nm using a heating rate of 0.5°C/min, a slit of 2 nm, and a response of 0.2 s. Tm values were obtained from the maxima of the first derivatives of the melting curves. Circular Dichroism spectra of the siRNA samples (1.0 *μ*M) were recorded at 15°C using a 1 cm quartz cell in the Jasco J-815 spectropolarimeter equipped with a PFD-425S thermal controller unit. ^31^P NMR spectrum was recorded at 161.98 MHz on a Bruker WM-400 spectrometer using 85% H_3_PO_4_ as external standard.

### 2.2. General Procedures for RNA Oligomers Synthesis

All oligomers were synthesized on ABI Expedite 8909 oligo synthesizer by using standard and modified protocols (1 *μ*M scale), 2′-OTBDMS-RNA phosphoramidite monomers, and standard RNA synthesis reagents. Unmodified RNAs** a** and** b **were prepared on SynBase CPG solid support (CPG-OH, loading 0.04 meq g^−1^) (a: calculated mass 6646,1, found 6645.9; b: calculated mass 6669.1, found 6667.8).

The RNA oligomers** C** and** D **were synthesized using last coupling monomer 5′-MMT-amino-T phosphoramidite. After deprotection (2% DCA in DCM), the 5′-amino group of oligomer was coupled to 2-(N-(2-((4-methoxyphenyl)diphenylmethoxy)ethyl)-2-(5-methyl-2,4-dioxo-3,4-dihydropyrimidin-1(2H)-yl)acetamido) acetic acid (0.2 M in DMF) [35] by using a solution 0.2 M HATUin DMF, 0.2 M DIPEA, and 0.3 M lutidine in DMF. The coupling cycle was carried out by a procedure slightly modified with respect to the standard PNA synthesis protocols in the steps of washing and deblocking as described here: (1) washing with 2.5 mL of (ACN/DMF) (1 : 1, v/v); (2) coupling 20 minutes; (3) washing with 2.5 mL of ACN/DMF (1 : 1, v/v); (4) capping with 5% of acetic anhydride and 6% of 2,6-lutidine in DMF, 2.0 mL; (5) washing with 2.5 mL of ACN/DMF (1 : 1, v/v) and 2.5 mL of DMF; (6) deblocking with a solution of dichloroacetic acid (2%) in ACN; (7) washing with 2.5 mL of DMF and 5 mL of ACN (C: calculated mass 6910.2, found 6910.0; D: calculated mass 7240.4, found 7238.0).

The RNA oligomers** E** and** F **were grown on the support** VIII,** following the same procedures described for the 5′ terminal modification of the oligomers** C** and** D** (E: calculated mass 6958.9, found 6957.7; F: calculated mass 7288.1, found 7286.9).

The RNA oligomers** G** and** H **were prepared after solid phase phosphorylation of the oligomers** C** and** D**. The phosphorylation reaction was achieved by using a solution 0.1 M of chemical phosphorylation reagent [36] in ACN with 0.3 M BTT in ACN. The oxidation was achieved by treatment with I_2_/H_2_O/Py (G: calculated mass 6990.2, found 6988.9; H: calculated mass 7320.3, found 7319.1).

The RNA oligomers** I** and** L **were synthesized on support** VIII, **following the same procedures described for 5′ terminal couplings and phosphorylation of the oligomers** G** and** H** (I: calculated mass 7038.9, found 7037.7; L: calculated mass 7368.1, found 7366.9).

The RNA oligomers** M** and** N **were prepared on standard SynBase CPG support, by introducing derivative** X **as last coupling monomer. The last coupling was carried out on automated synthesizer with a 0.1 M solution of derivative** X **in ACN (double coupling) and by using 0.3 M BTT in ACN, as activating solution. All succeeding procedures followed the standard protocol for RNA synthesis (M: calculated mass 7004.1, found 7002.8; N: calculated mass 7334.3, found 7333.1).

The RNA oligomers** O** and** P **were grown on support** VIII**, following the standard protocol for RNA synthesis (O: calculated mass 6693.8, found 6692.6; P: calculated mass 6716.8, found 6715.0).

### 2.3. Synthesis of Derivative VI

572 mg of SynBase CPG solid supports (CPG-OH, high loading 0.10 meq g^−1^) were functionalized with 297 mg (1.0 mmol) of Fmoc-Gly-OH using PyBOP (520 mg, 1.0 mmol) and HOBT (153 mg, 1.0 mmol) as activating agents in dry DMF (6 mL). The mixture was kept at r. t. for 16 h under shaking. The support was filtered and washed with DMF and Et_2_O and then dried under reduced pressure, thus obtaining support** III** (0.089 mmol/g, 89% coupling yield). The yield of incorporation of the glycine residue was calculated by quantitative UV measurements (301 nm) of the fluorene derivative released by piperidine/DMF (1 : 4, v/v) treatment of weighed amounts of support. Gly-linking support was washed with pyridine and then treated with acetic anhydride in pyridine (6.0 mL, 2 : 3, v/v, 1 h, r.t.) to block the unreacted hydroxy groups. After deblocking of amino function of supported glycine by a solution of piperidine in DMF (20%), 2-(N-(2-((4-methoxyphenyl)diphenylmethoxy)ethyl)-2-(5-methyl-2,4-dioxo-3,4-dihydropyrimidin-1(2H)-yl)acetamido) acetic acid (0.2 M in DMF) [35] was coupled by a solution 0.2 M HATUin DMF, 0.2 M DIPEA, and 0.3 M lutidine in DMF. The coupling cycle was carried out by a slightly modified procedure consisting of the following steps: (1) washing with 2.5 mL of (ACN/DMF) (1 : 1, v/v); (2) coupling 20 minutes; (3) washing with 2.5 mL of ACN/DMF (1 : 1, v/v); (4) capping with 5% of acetic anhydride and 6% of 2,6-lutidine in DMF, 2.0 mL; (5) washing with 2.5 mL of ACN/DMF (1 : 1, v/v) and 2.5 mL of DMF; (6) deblocking with a solution of dichloroacetic acid (2%) in DCA; (7) washing with 2.5 mL of DMF and 5 mL of ACN (0.089 meq g^−1^, 100% coupling yield). The phosphorylation reaction was achieved (0.088 meq g^−1^, 99% coupling yield) by using a solution 0.1 M of chemical phosphorylation reagent in ACN with 0.3 M BTT in ACN. The oxidation was achieved by treatment with I_2_/H_2_O/Py. After ammonium hydroxide treatment and purification by C18 Sep-Pak columns eluted by water/ACN (7 : 3, v : v) derivative** VI** was isolated and characterized (ESI-MS: m/z = 940.267 [M-2H^+^+2Na^+^], calculated for C_41_H_49_N_6_O_15_P: 896,30; ^31^P NMR *δ*: 1.846).

### 2.4. Functionalization of the CPG Resin: Support VIII

Fmoc-Gly-OH (60 mg, 0.2 mmol), PyBop (104 mg, 0.2 mmol), and HOBt (30 mg, 0.2 mmol) in dry DMF (5.0 mL) were added to CPG-OH resin (500 mg, 0.04 meq g^−1^ of hydroxy groups), previously washed with DMF. The mixture was kept at r.t. with shaking for 24 h. The support was filtered and washed with DMF and Et_2_O and then dried under reduced pressure, thus providing functionalized CPG (0.035 mmol/g, 89% coupling yield). The yield of incorporation of the glycine residue was calculated by quantitative UV measurements (301 nm) of the fluorene derivative released by piperidine/DMF (1 : 4, v/v) treatment of weighed amounts of support. Gly-linking support was washed with pyridine and then treated with acetic anhydride in pyridine (6.0 mL, 2 : 3, v/v, 10 min, r.t.) to block the unreacted hydroxy groups. After deblocking of amino function of supported glycine by a solution of piperidine in DMF (20%), N-(Thymin-1-ylacetyl)-N-(2-Fmoc-aminoethyl)glycine (53 mg, 0.2 mmol) was reacted in DMF and in presence of HATU (76 mg, 0.2 mmol) and DIPEA (52 *μ*L, 0.3 mmol) for 1 h at r.t. with shaking. The coupling yields, measured as previously described, were 90% (0.031 mmol/g). A capping procedure was carried out, after washing with DMF, by the addition of acetic anhydride in pyridine (2 : 3, v/v, 1 h, r.t.). After deblocking of amino function of supported PNA by a solution of piperidine in DMF (20%), 2-(N-(2-((4-methoxyphenyl)diphenylmethoxy)ethyl)-2-(5-methyl-2,4-dioxo-3,4-dihydropyrimidin-1(2H)-yl)acetamido) acetic acid [35] (0.2 M in DMF) was coupled by a solution 0.2 M HATUin DMF, 0.2 M DIPEA, and 0.3 M lutidine in DMF. The coupling cycle was carried out by a slightly modified procedure consisting in the following steps: (1) washing with 2.5 mL of (ACN/DMF) (1 : 1, v/v); (2) coupling 20 minutes; (3) washing with 2.5 mL of ACN/DMF (1 : 1, v/v); (4) capping with 5% of acetic anhydride and 6% of 2,6-lutidine in DMF, 2.0 mL; (5) washing with 2.5 mL of ACN/DMF (1 : 1, v/v) and 2.5 mL of DMF. The obtained support** VIII** resulted in 0.031 meq g^−1^ (100% coupling yield) as judged by quantitative UV measurements (478 nm) of the MMT cation released by dichloroacetic acid (2%) in DCM treatment of weighed amounts of** VIII**.

### 2.5. Synthesis of Derivative X

Methyl ester derivative of* hegPNA* **IX** (0.50 g; 1.67 mmol) was dissolved in anhydrous DCM (15 mL). Anhydrous DIEA (0.63 mL, 3.69 mmol) and 2-cyanoethyl-N,N-diisopropylphosphoramidochloridite (0.24 mL; 1.10 mmol) were added to this solution. After 1 h the solvent was evaporated* in vacuo* and the residue dissolved in EtOAc and washed four times with brine. The organic phase was dried and evaporated* in vacuo*. The residue was purified by column chromatography on silica gel using DCM/EtOAc/N_3_Et, (49.5 : 49.5 : 1, v : v : v) as the eluent. 0.65 g (78% yield) of derivative** X** was obtained (^31^P-NMR (CDCl_3_) *δ*: 146.0, 146.7).

### 2.6. Cell Cultures, Transfections, and Luciferase Assay

HeLa cells were grown at 37°C, 5% CO_2_ in Dulbecco's modified Eagle's medium supplemented with 10% fetal bovine serum (FBS) (EuroClone), 100 units mL^−1^ penicillin, and 100 mg mL^−1^ streptomycin (EuroClone). The day before transfection, cells were trypsinized, diluted in the appropriate amount of growth medium without antibiotics, and transferred to 12-well plates (1 mL per well) such that they were 80–90% confluent at the time of transfection. Cotransfections of reporter plasmids (per well 1 *μ*g pGL2-control, encoding the firefly* Photinus pyralis* luciferase and 0.05 *μ*g phRL-TK, encoding* Renilla reniformis* luciferase, Promega) and 10 nM siRNAs were carried out with Lipofectamine2000 (Invitrogen) as described by the manufacturer. Luciferase activities were monitored 2 days after transfection using Dual-Luciferase Reporter Assay System (Promega) according to the manufacturer's protocol. The firefly luciferase activity was normalized to the Renilla luciferase activity and the uninhibited activity (plasmids encoding the luciferases cotransfected with unrelated siRNA as control) was set to 1. Data represent mean normalized luciferase activity from at least three experiments ± s.d.

### 2.7. Stability of siRNAs in 100% FBS

7.5 *μ*L of unmodified and modified siRNAs (20 *μ*M) were incubated with 75 *μ*L of FBS at 37°C. Aliquots of 22 *μ*L (40 pmoles) were withdrawn at different time points and immediately frozen. The solutions were then extracted with phenol and siRNAs were precipitated with ethanol. Samples were subjected to electrophoresis in 15% polyacrylamide-tris-borate-EDTA (TBE) under nondenaturing conditions and visualized by ethidium bromide staining. Equal amounts of siRNAs before serum incubation were extracted with phenol and loaded as controls. Gel images were captured by ChemiDoc XRS (Bio-Rad) and RNA electrophoretic bands were quantified by Image Lab software (Bio-Rad). Signal intensity value of the intact siRNA was set at 1.

### 2.8. Molecular Docking

The miR-20a/hAgo 2 three-dimensional structure was downloaded from the Protein Data Bank (PDB code 4F3T) [9]. The co-crystal RNA present in this structure was used as template for the construction of the modified siRNAs featuring the modifications at the 5' and 3' end (viz. AS, antisense strand, of siRNAs** 16** and** 17**, resp.,). SiRNA structures were built using the builder in the Maestro package of Schroedinger Suite 2010 and optimized using a version of MacroModel also included. The following parameters of energy minimization were used: OPLS2005 force field was used, water was used as an implicit solvent, and a maximum of 5000 iterations of the Polak-Ribier conjugate gradient minimization method was used with a convergence threshold of 0.01 kJ mol^−1^ Å^−1^.

The new version of the docking program AutoDock4.2 (AD4) [37, 38], as implemented through the graphical user interface called AutoDockTools (ADT), was used to dock these siRNAs. The constructed compounds and the receptor structure were converted to AD4 format files using ADT generating automatically all other atom values. Since AD4 has a limit in the number of ligand rotatable bonds only the PNAs (viz. dTdTa, dTdTb, and dTdTc) were free to rotate. The docking area was centered around the putative binding site (PAZ domain for** 17** and MID domain for** 16**). A set of grids of 90 Å × 40 Å × 75 Å with 0.375 Å spacing was calculated around the docking area for the ligand atom types using AutoGrid4.2. For each ligand, 700 separate docking calculations were performed. Each docking calculation consisted of 25 million energy evaluations using the Lamarckian genetic algorithm local search (GALS) method. The GALS method evaluates a population of possible docking solutions and propagates the most successful individuals from each generation into the subsequent generation of possible solutions. A low-frequency local search according to the method of Solis and Wets is applied to docking trials to ensure that the final solution represents a local minimum. All dockings described in this paper were performed with a population size of 250, and 300 rounds of Solis and Wets local search were applied with a probability of 0.06. A mutation rate of 0.02 and a crossover rate of 0.8 were used to generate new docking trials for subsequent generations, and the best individual from each generation was propagated over the next generation. The docking results from each of the 700 calculations were clustered on the basis of root-mean square deviation (rmsd) (solutions differing by less than 3.0 Å) between the cartesian coordinates of the atoms and were ranked on the basis of free energy of binding (ΔG_AD4_). These lowest energy conformations were visually inspected for good chemical geometry. Because AD4 does not perform any structural optimization and energy minimization of the complexes found, a molecular mechanics/energy minimization (MM/EM) approach was applied to refine the AD4 output using the Schroedinger Suite 2010. The computational protocol applied consisted of the application of 100000 steps of the Polak-Ribiére conjugate gradients (PRCG) or until the derivative convergence was 0.05 kJ/mol. All complexes pictures were rendered employing the UCSF Chimera software [39].

## 3. Results

### 3.1. Chemistry

#### 3.1.1. Synthesis of Unmodified siRNA 1 and Modified siRNAs 2–4

**Figure 2:**
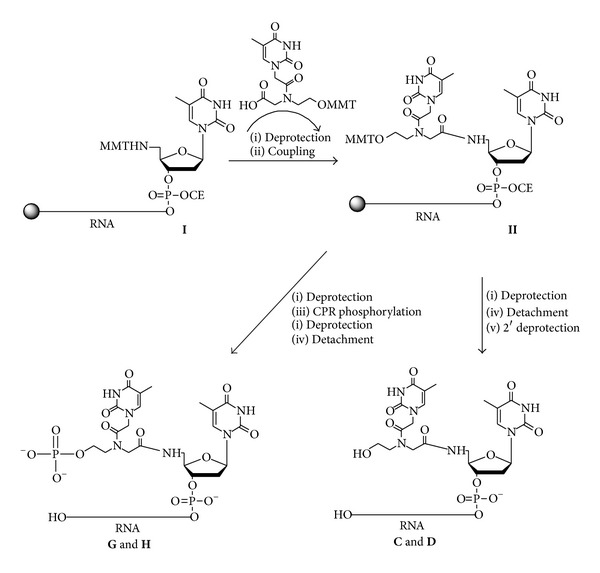
Assembling of the RNA oligomers** C**,** D**,** G,** and** H**. Reagents and conditions: (i) 2% DCA in DCM; (ii) 0.2 M HATU in DIPEA/DMF; (iii) 0.1 M CPR in ACN, 0.3 M BTT in ACN; (iv) NH_4_OH, 50°C, 12 h; (v) Et_3_N·3HF in DMF 1 : 3 v/v, 65°C, 150 min.

In the present work our attention was mainly focused on specific chemical modification at 5′ region of the antisense and sense strands of siRNAs. In this respect, we decided to modify an siRNA targeting firefly luciferase mRNA [2] because of the possibility of testing it by an automated luciferase assay allowing an easy and accurate judgment of the gene silencing activity in cultured mammalian cells, thus comparing chemically modified siRNAs to the unmodified one. Unmodified RNA strands** a-b** in Table 1 (antisense 5′-UCGAAGUAUUCCGCGUACGTT-3′ and sense 5′-CGUACGCGGAAUACUUCGATT-3′) were synthesized following DNA/RNA fully automated protocols. The synthesis of the RNA-5′-PNA-OH** C-D** was carried out on CPG-OH support using commercially available 2′-O-TBDMS-3′-phosphoramidite ribonucleotides and 5′-MMT-NH-thymidine-3′-O-phosphoramidite. MMT-hydroxyethylglycine PNA monomers (MMT-O-PNA) were synthesized following reported procedures and characterized by ^1^H and ^13^C NMR spectroscopies [35]. The synthesis of the RNA tract was performed on a RNA synthesizer and 5′-MMT-NH-thymidine-3′-O-phosphoramidite was introduced as last coupling monomer to obtain support** I** (Figure 2). In order to incorporate the PNA monomer at 5′ end of RNA sequence (support** II,** Figure 2), MMT-hydroxyethylglycine PNA monomer was coupled using PNA standard procedure (HATU in DIPEA/DMF).

After deblocking of the hydroxyl function by 2% DCA/DCM treatment, the oligomers were cleaved from the support by treatment with NH_4_OH at 50°C overnight. The 2′-O-TBDMS protecting group was removed by Et_3_N·3HF and the resulting fully deprotected RNA oligomers** C** and** D** (Figure 2) were precipitated from ethanol, purified by anion exchange and RP-HPLC, and characterized by MALDI-TOF mass spectrometry. The MS-data confirmed the identity of the synthesized oligomers. Then, the oligomers** a**,** b**,** C, **and** D **were combined to form siRNAs** 1–4** (Table 1) following the annealing procedure as described above.

#### 3.1.2. Synthesis of Modified siRNAs 8–10: Solid Phase Chemical Phosphorylation

**Figure 3:**
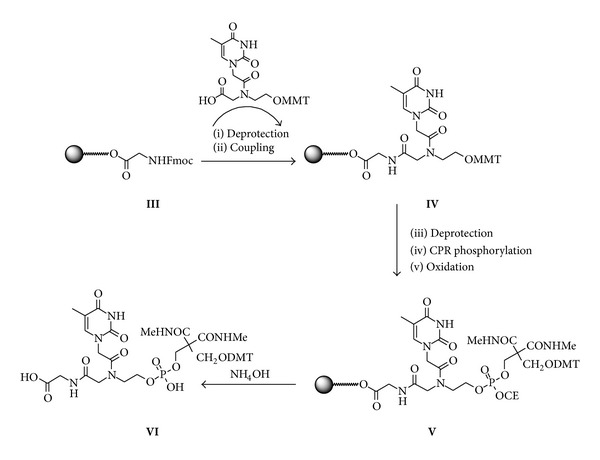
Solid phase synthesis of the derivative** VI**. Reagents and conditions: (i) 20% Piperidine in DMF; (ii) 0.2 M HATU in DMF, 0.2 M DIPEA, 0.3 M Lutidine in DMF; (iii) 2% DCA in DCM; (iv) 0.1 M CPR in ACN, 0.3 M BTT in ACN; (v) I_2_/H_2_O/pyridine.

Chemical phosphorylation of the 5′-terminus of oligonucleotides is routinely achieved, with high yields using the CPR ([3-(4,4′-Dimetoxytrityloxy)-2,2-(N-methylamidocarbonyl)]-propyl-[(cyanoethyl)-(N,N-diisopropyl)]phosphoramidite) [36]. This method requires CPR introduced by coupling with phosphoramidite method. Aside from its inherent convenience, CPR also has the advantage over enzymatic methods in allowing immediate determination of the phosphorylation efficiency due to the presence of the DMT protecting group. The synthesis of the RNA-5′-PNA-O-phosphate** G-H** required a previous phase to study and optimize the chemical phosphorylation of* heg*PNA on solid phase. For this purpose, the chemical phosphorylation of* heg*PNA monomer was tested on CPG-OH support. The solid supports were functionalized with a Fmoc-Gly-OH as spacer using PyBop/HOBT as activating agents. The yield of glycine introduction, evaluated by spectrophotometric measurements of fluorene derivative, was 0.035 meq/g. CPG-Gly derivatized support was therefore functionalized with the MMT protected* hegPNA* monomer by PNA coupling procedure (HATU/DIPEA in DMF). After MMT removal, the OH function was chemically phosphorylated by using commercially available CPR. The CPG support gave good results in the phosphorylation reaction. In fact, the yields of phosphorylation on CPG support resulted 99% as calculated by the spectrophotometric measurements of DMT cation released after deprotection of phosphate in DCA/DCM. After basic treatment for deprotection and detachment from support, PNA-O-phosphate derivative** VI** (Figure 3) was purified by RP-C18 cartridge and characterized by ^31^P NMR and ESI-MS. On the basis of these results, CPG support was chosen to synthesize RNA-5′-PNA-O-phosphate** G-H (** Figure 2 **)**. The identity of full-length oligomers was confirmed by MALDI-TOF analysis. The oligomers** a**,** b**,** G,** and** H **(Figure 2) were combined to form siRNAs** 8–10** (Table 1) following the annealing procedure as described above.

#### 3.1.3. Synthesis of Modified siRNAs 5–7, 11–13, and 17–19

**Figure 4:**
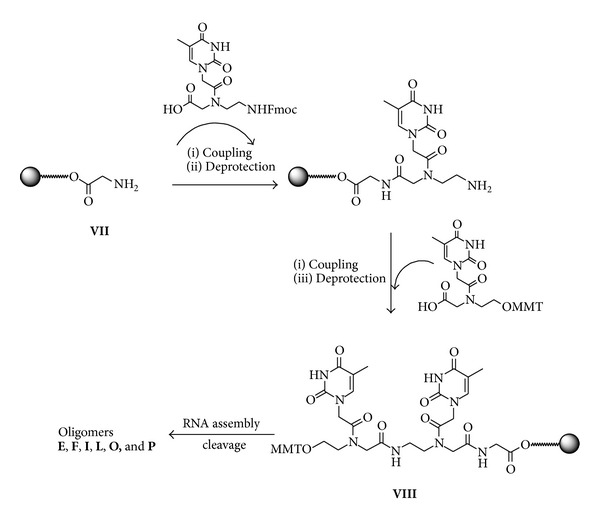
Synthesis of the support** VIII.** Reagents and conditions: (i) 0.2 M HATU in DIPEA/DMF; (ii) 20% Piperidine in DMF; (iii) 3%; DCA in DCM.

Modified 3′,5′-PNA siRNAs were synthesized in order to study the effect of PNA on both 5′ and 3′ ends of siRNAs sense and antisense strands. The support** VIII** (Figure 4) was synthesized starting from CPG-OH support** VII** by reaction with Fmoc-amino and MMT-hydroxyethylglycine PNAs following procedures reported elsewhere [27]. After deprotection of OH function, the synthesis of RNA tract was performed using commercially available 2′-O-TBDMS-3′-O-phosphoramidite ribonucleotides on oligo synthesizer affording oligomers** O-P** (Table 1). To obtain the oligomers** E-F **and** I-L **(Table 1), the last couplings were done with 5′-MMT-NH-thymidine-3′-O-phosphoramidite and MMT-O-PNA monomer as described for** C-D** and** G-H**. Deprotection and cleavage of oligomers from support were carried out as described above. HPLC purification and MS-data confirmed the identities of chimeras** E-F**,** I-L, **and** O-P**. The siRNAs** 5–7**,** 11–13,** and** 17–19** were assembled as described in Table 1 following the annealing procedure as described above.

#### 3.1.4. Synthesis of the siRNAs 14–16

**Figure 5:**
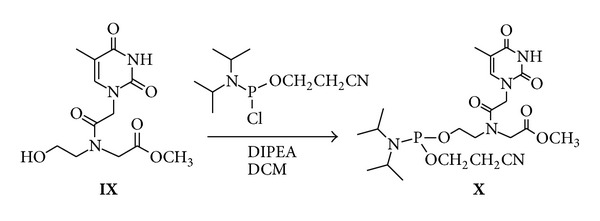
Synthesis of phosphoramidite PNA building block** X**.

We synthesized a new set of siRNAs bearing at 5′ end a PNA monomer linked by phosphodiester bridge and having the C-terminus as methylamide cap. For this purpose, phosphoramidite PNA building block** X **(Figure 5) was prepared by reaction of methyl ester derivative of* hegPNA* **IX** with 2-cianoethyl-N,N-diisopropylchlorophosphoramidite in dry DIPEA/DCM. After LC purification and ^31^P NMR characterization, the PNA phosphoramidite derivative** X **was used as last coupling monomer to obtain the precursor of the oligomers** M-N**.

Methylamine treatment (33% in absolute ethanol) accomplished the deprotection and detachment of the oligomers from the support as methylamide derivatives. Desilylation gave the RNA-5′-O-PNA-CONHCH_3_ oligomers** M-N **(Table 1) that after purification and characterization by MALDI-TOF MS were combined with** a-b** to give the siRNAs** 14–16** (Table 1) following the annealing procedure as described above.

### 3.2. Circular Dichroism Characterization of Native and Modified siRNAs

Although the conformational features alone do not warrant an interfering activity, a double-helical RNA-like A conformation is required for effective gene silencing. Indeed, the modified siRNAs** 2–19** were all found to retain an RNA-like A conformation exhibiting CD spectra similar to those of the unmodified siRNA** 1** with a typical positive shoulder at 280 nm and a large positive band at 260 nm. This behavior suggested that terminal amide linkages at 3′ and/or 5′ ends do not interfere with the typical A conformation of dsRNA. Thermal stability of the siRNAs** 2–19** was also considered by thermal denaturation CD experiments highlighting a general thermodynamic destabilization for all modified duplexes. In fact, melting temperatures lower than that of siRNA** 1** (76°C) were recorded in all cases suggesting that the presence of the terminal amide linkages caused a limited destabilization of the secondary structure, as inferred from CD-melting values (Table 1).

### 3.3. Compatibility of Modified siRNAs with the RNAi Machinery

**Figure 6:**
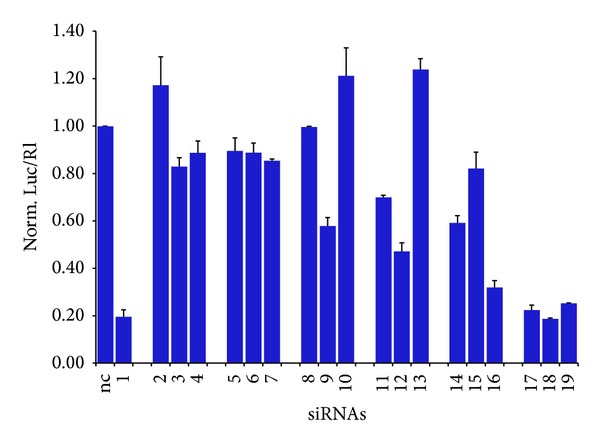
RNAi activity of native and modified siRNAs toward luciferase gene expression in HeLa cells. Small interfering RNAs were transfected at 10 nM concentration and luciferase activities were determined after 48 h. The firefly luciferase activity (Luc) was then normalized to that of Renilla luciferase (Rl) and the uninhibited activity of cells transfected with an unrelated siRNA (nc) was set to 1. Data are the mean ± s.d. from at least three experiments.

To verify the compatibility of modified siRNAs containing amide linkages with the cellular RNAi machinery, siRNAs** 2–19** were evaluated for their ability to specifically inhibit firefly luciferase gene expression in cultured HeLa cells. siRNA duplexes were cotransfected with the reporter plasmid combination pGL2/phRL-TK using cationic liposomes. pGL2 is transcribed into the* Photinus pyralis* luciferase mRNA, targeted by the siRNA, whereas phRL-TK encodes the* Renilla reniformis* luciferase, used as control to normalize the data with the efficiency of transfection. As shown in Figure 6, the native siRNA** 1** effectively and selectively reduced firefly luciferase activity by more than 80% compared to an unrelated siRNA (nc).

We then confirmed that the introduction of PNA modification at the 3′end in either or both strands does not show any loss of interfering effect, because all the modified duplexes (siRNAs** 17–19**) were as efficient as the siRNA** 1**. On the other hand, the presence of the amide linkage dramatically decreased the interference activity when it was located at 5′ and at both 3′, 5′ regions of siRNAs, demonstrating that the modification at 5′ end mostly controls the silencing activity. Interestingly, “5′-capped” siRNAs partially restored the interfering effect. Therefore, the siRNA** 16** appeared to be more effective than all the other 5′-modified duplexes, while the siRNA** 18** appeared to be more effective than all the other modified duplexes.

### 3.4. Serum Stability of Modified siRNAs

**Figure 7:**
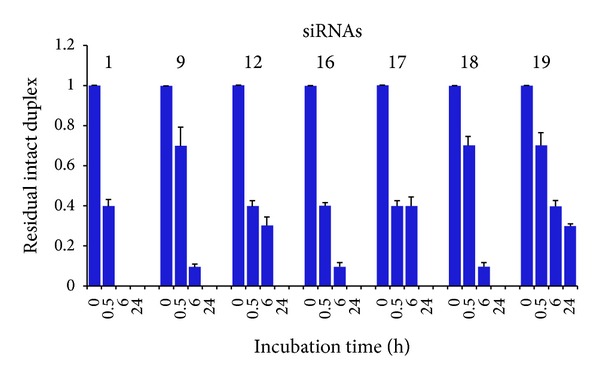
Stability of natural and modified siRNAs in 100% fetal bovine serum at 37°C. After incubation, siRNAs were analyzed by PAGE and ethidium bromide staining. Gel images were captured by ChemiDoc XRS (Bio-Rad) and RNA bands were quantified by Image Lab software (Bio-Rad). Signal intensity values at* t*_*0*_ were set at 1.

Improving the biostability is also crucial for therapeutic purposes of synthetic siRNAs. Experiments were planned to test whether the introduction of the amide linkages at 5′ and/or at 3′ ends leads to siRNAs more stable than unmodified ones in the extracellular environment. Therefore, the nuclease resistance of** 2–19** was investigated through incubation in 100% fetal bovine serum (FBS) at 37°C using unmodified siRNA** 1** as control (Figure 7). At various incubation times, aliquots of each siRNA were analyzed by electrophoresis on 15% polyacrylamide gels to detect any degradation products. The unmodified siRNA** 1** was greatly degraded within 30 min (Figure 7) as well as almost all siRNAs featuring only one modified strand (data not shown), except for** 9** and** 12** that exhibited significant amounts of intact duplex at 6 h of incubation. Similar results were obtained with siRNAs** 16–18**. Finally, the introduction of PNA on both strands markedly increased the resistance to serum-derived nucleases as demonstrated by** 19** that showed still evident intact duplex even after 24 h of incubation.

### 3.5. Molecular Docking Simulations

Molecular docking simulations were attained in order to provide a putative model of the interaction of the newly identified siRNAs with the human Argonaute 2 (hAgo 2) protein at atomistic level. In particular, the software AutoDock4.2 (AD4) [37, 38] was employed to dock the ASs of the siRNAs bearing the most performing modifications at the 3′ and 5′ ends. In particular, the chimeric 3′-OH-PNA AS of siRNA** 17** (oligomer O) and the AS of "5′-capped" siRNA** 16** (oligomer M)” were docked in the recently published structure of hAgo 2 in complex with miR-20a (PDB code 4F3T) [9]. The modified siRNA ASs were constructed starting from the RNA cocrystal conformation where each nucleotide was mutated to obtain the target sequence, while at the 3′ and 5′ terminals the proper modifications were attained. These latter groups were free to move during the docking simulations. For both docking calculations only the lowest energy conformations (ΔG_AD4_) were considered and analyzed.

**Figure 8:**
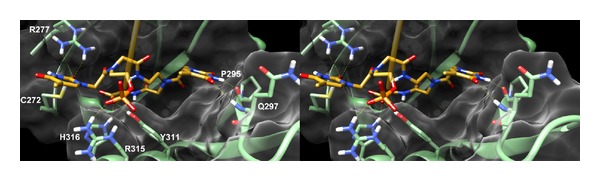
Stereoview of the predicted binding mode of the 3′-OH-PNA AS of siRNA** 17** in the hAgo 2 X-ray structure. For clarity reasons, the PNA structure is depicted (yellow sticks) while the RNA backbone is shown as an orange transparent ribbon. The protein is depicted as green sticks (interacting residues) and ribbons and white transparent surface. H-bonds are indicated as yellow dashed lined.

In the theoretical complex between the AS of siRNA** 17** and hAgo 2 the modified RNA adopted a conformation similar to that of the cocrystal RNA with the 3′ end contacting the protein PAZ domain. In this position, the terminal PNA residues are embedded in a wide gorge of the aforementioned protein region establishing several favorable interactions (Figure 8). In particular the phosphate group linking the PNA structure to the RNA one is forming an ionic interaction with R315 while the* heg* residue is able to project the attached thymine base to form two H-bonds with R277 and C272. On the other hand, the second thymine base is pointing towards the opposite site of the gorge H-bonding with P295 and Q297 backbone CO and NH, respectively. Finally, the terminal carboxylate group is able to H-bond H316 and Y311 side-chains.

**Figure 9:**
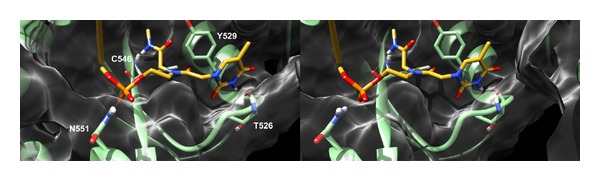
Stereoview of the predicted binding mode of the “5′-capped” AS of siRNA** 16** in the hAgo 2 X-ray structure. For clarity reasons, only the PNA structure is depicted (yellow sticks) while the RNA backbone is shown as an orange transparent ribbon. The protein is depicted as green sticks (interacting residues) and ribbons and white transparent surface. H-bonds are indicated as yellow dashed lined.

A well-defined binding pose was also found for the “5′-capped” AS of siRNA 16 where the terminal 5′ is pointing towards the hAgo 2 middomain (Figure 9). In this position the phosphate group is H-bonding N551 with the thymine moiety being optimally oriented to establish a *π*-*π* interaction with Y529 and an H-bond with T526 backbone CO. Moreover, the terminal methylamide cap can form an additional H-bond interaction with C546 backbone CO.

## 4. Discussion

A rational design of effective chemically modified siRNAs must consider the 3′ and 5′ termini of siRNAs as critical determinants for the silencing activity because these regions interact with the binding domains of hAgo 2, that is, the signature component of the RNAi machinery. In particular, the selective loading of the guide strand into RISC is essential for avoiding undesirable side effects, while the release of the 3′ end from the PAZ domain during the cleavage of the mRNA is crucial to perform RNase activity [40]. In this respect, experiments on cell cultures have demonstrated that chemical modifications of siRNAs at 3′ end modulate the silencing activity, increase the nuclease resistance, and influence the affinity of the 3′-overhang portion for PAZ [41–44]. Instead, the phosphorylation of the 5′ end of siRNA is one of the first steps required for antisense strand selection and for its function in RISC [45, 46]. The 5′ end of siRNA is tethered to hAgo 2 through a multitude of interactions to form a very tight binding pocket composed of residues mostly from the middomain and capped on one side by PIWI domain residues [47–50]. All three nonbridging oxygen atoms of the 5′ phosphate interact with several protein side chains and it has been suggested that the proper interactions between the 5′-phosphate and hAgo 2 define the position of the RNA guide with respect to the active site to ensure that cleavage of targets occurs at a well-defined and predictable position. In addition, early work on siRNA chemical modifications suggested the importance of the thermal stability of the siRNA duplex, as measured by the melting temperature, Tm, on the gene silencing activity [51–53]. However, there is no obvious correlation between the overall duplex Tm and the gene silencing activity of the siRNA. Rather, specific regions of the siRNA duplex have distinct tolerances toward stabilization and destabilization, resulting in position-specific changes of activity upon incorporation of chemical modifications that affect thermal stability [54]. Therefore, the thermodynamic properties of siRNA molecules play a central role in determining the molecule's functionality by facilitating several steps, namely, duplex unwinding, correct strand selection, reduced unspecific recognition of target, and mRNA turnover.

SiRNAs** 2–7**, bearing* heg*PNA at 5′ end of sense and antisense strands, did not exhibit any mRNA degradation activity and it might be postulated that in these siRNAs the* heg*PNA inhibits phosphorylation event at the 5′ terminus. Thus, the absence of the phosphate at 5′ end of guide strand in the siRNAs** 2** and** 5** could result in a loss of specificity and efficiency of target cleavage because the RNA guide might adopt incorrect positions in the RNA binding groove of hAgo 2. This is similar to previously published results [55] where unlocked nucleic acid (UNA) modification at positions 1 and 2 of 5′-terminus prevented phosphorylation by Clp1 kinase abrogating interaction with hAgo 2. Furthermore, it is also reasonable that in siRNAs** 2** and** 5, **the amide-bond linkage placed in substitution of the phosphodiester bridge between the nucleotides 1 and 2 of the 5′ region might derange the required interactions between the AS strand and hAgo 2. Modifying siRNAs by introducing* heg*PNA monomer at the 5′-terminus of the sense strand (siRNAs** 3** and** 6**) resulted in a loss of silencing activity. This unexpected result could be explained by the presence of a thermal destabilization due to the structural complexity induced by the PNA in the siRNAs** 3** and** 6**, as confirmed by thermal denaturation studies (Table 1). In this respect, the thermal destabilization would translate in the SS selection by RISC rather the AS and this is consistent with the asymmetry rule of siRNAs. This hypothesis is also confirmed by the observed behavior of siRNAs** 4** and** 7** where the introduction of PNA at 5′ end of both strands resulted again in a loss of silencing activity. Noteworthy, the presence of PNA also at 3′ end (siRNAs** 5–7**) had no improvement on the silencing activity, although the analysis of data resulting from silencing activity of the siRNAs** 17–19** showed that the PNA units into 3′-dangling region were well tolerated by RNAi machinery. In particular, the modification of the sense strand of siRNA** 18** resulted in an increased silencing activity probably by inducing the correct loading of antisense strand by RISC. In this respect, the theoretical model of the complex between the AS of** 17** and the PAZ domain of hAgo 2 would indicate that, in this siRNA, the 3′-OH-PNA is able to establish several and tight interactions with the protein counterpart and thus positively modulating the rates of dislodging and lodging of the AS in and from the PAZ domain and providing, in turn, a further determining factor for its efficiency and potency.

In order to assess the impact of the chemical phosphorylation of* heg*PNA at 5′ end on the interfering activity, siRNAs** 8–13** were synthesized. As shown in Figure 5, siRNAs** 8 **and** 11** containing a PNA-phosphate at the 5′ end of antisense strand were less potent than unmodified siRNA, reproducing the same results obtained for siRNAs** 2** and** 5**. In addition to the lack of the proper AS/hAgo 2 interactions ascribable to the presence of the internucleotidic amide-bond linkage, the low silencing potential may be also caused by the 5′-terminal phosphate instability. In principle, the 5′-phosphate could be removed in the cellular environment and the 5′ terminal* heg*PNA could not be rephosphorylated by cellular kinase Clp1 as already suggested from the behavior of the siRNAs** 2–7**. Furthermore, siRNAs** 9** and** 12** resulted in being significantly more active than siRNAs** 8** and** 11. **The only partial restoring of the silencing activity of** 9** and** 12** may be rationalized considering that both the modified SS (no silencing) and the unmodified AS (silencing) occur.

Finally, we synthesized novel modified siRNAs bearing an* heg*PNA monomer inserted at 5′ end of RNA domain by phosphodiester linkage and having at the C-terminus a methylamide function (siRNA** 14–16**, Table 1). Interestingly, improved silencing performances were observed for siRNAs** 14** and** 16 **in whichwe supposethat the modified ASis selectively loaded on RISC due to the thermal asymmetry conferred by methylamide PNA at 5′ end. In fact, siRNA** 15 **was not effective, probably, since the modified sense strand is preferentially selected. The “5′-capped” siRNAs** 14** and** 16** partially restored an interfering effect besides methylamide cap blocks the siRNA versus phosphorylation [56–61]. Also, the specific interaction detected between the methylamide and the hAgo 2 middomain would further substantiate a role of the chemical modification in the selective loading of the AS by the protein. Furthermore, it is worth mentioning that the presence of phosphodiester linkage between residues 1 and 2 in the 5′ region of the siRNAs** 14** and** 16 **seems to have an additional role in maintaining the correct binding to direct mRNA degradation.

## 5. Conclusions

In summary, we reported the synthesis of modified siRNAs containing terminal amide linkages by introducing* heg*PNA moieties at 5′, 3′, and at both termini. It was revealed that modified siRNAs are only compatible with the RNAi machinery when amide bonds are introduced at 3′ end of the siRNAs. In particular, poor efficacy was observed when* heg*PNA or* heg*PNA phosphate moieties are placed at 5′ ends because this region plays a crucial role in maintaining the correct binding to address the mRNA-target degradation. Further, “5′-capped” siRNAs partially restored the interfering activity maybe favoring the AS selective loading as well as the correct binding of the AS by the protein. Besides, we examined the biostability of the modified siRNAs and showed the introduction of amide linkages on both strands of siRNAs markedly increased the resistance to serum-derived nucleases. Molecular docking simulations were attained to give at atomistic level a clearer picture of the effect of the most interesting modifications on the interactions with the human Argonaute 2 PAZ, MID, and PIWI domains. In conclusion, this report appends further on the understanding of the effects of chemical modifications and of their location in siRNA structure on the silencing properties and sensitivity to the degradation by ribonucleases and thus opens to the future possibility to exploit these chemical modifications in studying new structure-function relationships.

The following abbreviations are used throughout the test.

## Figures and Tables

**Table 1:** 

	siRNA sequence	siRNAs	Tm °C	Δ
**a**	AS 3′*TT*GCAUGCGCCUUAUGAAGCU5′	siRNA **1** wt	**76**	**—**
**b**	SS 5′CGUACGCGGAAUACUUCGA*TT*3′			
**C**	3′*TT*GCAUGCGCCUUAUGAAGCT^NHCO^t^OH^5′	siRNA **2**	**65**	**−11**
**b**	5′CGUACGCGGAAUACUUCGA*TT*3′			
**a**	3′*TT*GCAUGCGCCUUAUGAAGCU5′	siRNA **3**	**65**	**−11**
**D**	5^′OH^t^CONH^TCGUACGCGGAAUACUUCGA*TT*3′			
**C**	3′*TT*GCAUGCGCCUUAUGAAGCT^NHCO^t^OH^5′	siRNA **4**	**65**	**−11**
**D**	5^′OH^t^CONH^TCGUACGCGGAAUACUUCGA*TT*3′			
**E**	3′*tt*GCAUGCGCCUUAUGAAGCT^NHCO^t^OH^5′	siRNA **5**	**66**	**−10**
**b**	5′CGUACGCGGAAUACUUCGA*TT*3′			
**a**	3′*TT*GCAUGCGCCUUAUGAAGCU5′	siRNA **6**	**66**	**−10**
**F**	5^′OH^t^CONH^TCGUACGCGGAAUACUUCGA*tt*3′			
**E**	3′*tt*GCAUGCGCCUUAUGAAGCT^NHCO^t^OH^5′	siRNA **7**	**66**	**−10**
**F**	5^′OH^t^CONH^TCGUACGCGGAAUACUUCGA*tt*3′			
**G**	3′*TT*GCAUGCGCCUUAUGAAGCT^NHCO^t^OP^5′	siRNA **8**	**67**	**−9**
**b**	5′CGUACGCGGAAUACUUCGA*TT*3′			
**a**	3′*TT*GCAUGCGCCUUAUGAAGCU5′	siRNA **9**	**66**	**−10**
**H**	5^′PO^t^CONH^TCGUACGCGGAAUACUUCGA*TT*3′			
**G**	3′*TT*GCAUGCGCCUUAUGAAGCT^NHCO^t^OP^5′	siRNA **10**	**67**	**−9**
**H**	5^′PO^t^CONH^TCGUACGCGGAAUACUUCGA*TT*3′			
**I**	3′*tt*GCAUGCGCCUUAUGAAGCT^NHCO^t^OP^5′	siRNA **11**	**66**	**−10**
**b**	5′CGUACGCGGAAUACUUCGA*TT*3′			
**a**	3′*TT*GCAUGCGCCUUAUGAAGCU5′	siRNA **12**	**68**	**−8**
**L**	5^′PO^t^CONH^TCGUACGCGGAAUACUUCGA*tt*3′			
**I**	3′*tt*GCAUGCGCCUUAUGAAGCT^NHCO^t^OP^5′	siRNA **13**	**66**	**−10**
**L**	5^′PO^t^CONH^TCGUACGCGGAAUACUUCGA*tt*3′			
**M**	3′TTGCAUGCGCCUUAUGAAGCTt^CONHCH_3_^5′	siRNA **14**	**70**	**−6**
**b**	5′CGUACGCGGAAUACUUCGA*TT*3′			
**a**	3′*TT*GCAUGCGCCUUAUGAAGCU5′	siRNA **15**	**68**	**−8**
**N**	5^′CH_3_NHCO^tTCGUACGCGGAAUACUUCGA*TT*3′			
**M**	3′TTGCAUGCGCCUUAUGAAGCTt^CONHCH_3_^5′	siRNA **16**	**70**	**−6**
**N**	5^′CH_3_HNCO^tTCGUACGCGGAAUACUUCGATT3′			
**O**	3′*tt*GCAUGCGCCUUAUGAAGCU5′	siRNA **17**	**76**	**—**
**b**	5′CGUACGCGGAAUACUUCGA*TT*3′			
**a**	3′*TT*GCAUGCGCCUUAUGAAGCU5′	siRNA **18**	**76**	**—**
**P**	5′CGUACGCGGAAUACUUCGA*tt*3′			
**O**	3′*tt*GCAUGCGCCUUAUGAAGCU5′	siRNA **19**	**76**	**—**
**P**	5′CGUACGCGGAAUACUUCGA*tt*3′			

## Abbreviations

BTT: 5-Benzylthio-1H-tetrazole
CD: Circular dichroism
CPG: Controlled pore glass
CPR: Chemical phosphorylation reagent
DCM: Dichloromethane
DCA: dichloroacetic acid
DEPC: Diethylpirocarbonate
DMF: N,N-Dimethylformamide
DMT: Dimethoxytrityl [bis(4-methoxyphenyl)(phenyl)methyl]
ESI: Electrospray ionization
FBS: Fetal bovine serum
Fmoc: 9-Fluorenylmethoxycarbonyl
HOBT: N-Hydroxybenzotriazole
HPLC: High performance liquid chromatography
MALDI-TOF: Matrix assisted laser desorption ionization-time of flight
PyBOP: (Benzotriazol-1-yloxy) tripyrrolidinophosphonium hexafluorophosphate
RNAi: RNA interference
RISC: RNA-induced silencing complex
RNase: Ribonuclease
RP-HPLC: Reverse phase HPLC
siRNA: Small interfering RNA
TBDMS: t-Butyldimethylsilyl
Tm: Melting temperature.
